# Rationale for reconsidering current regulations restricting use of hybrids in orange juice

**DOI:** 10.1038/s41438-020-0277-5

**Published:** 2020-03-07

**Authors:** Ed Stover, Frederick G. Gmitter, Jude Grosser, Elizabeth Baldwin, Guohong Albert Wu, Jinhe Bai, Yu Wang, Peter Chaires, Juan Carlos Motamayor

**Affiliations:** 1 USDA/ARS, US Horticultural Research Lab., 2001 S Rock Rd., Ft. Pierce, FL 34945 USA; 2 Citrus Research and Education Center, Univ. Florida, 700 Experiment Station Rd., Lake Alfred, FL 33850 USA; 3 US Department of Energy, Joint Genome Institute, Lawrence Berkeley National Laboratory, 1 Cyclotron Road, Berkeley, CA 94720 USA; 4 New Varieties Development & Management Corp., PO Box 941058, Maitland, FL 32794 USA; 5 The Coca-Cola Company, 2651 Orange Ave., Apopka, FL 32703 USA

**Keywords:** Plant breeding, Natural variation in plants

## Abstract

Huanglongbing (HLB) is a disease that has devastated the Florida citrus industry, threatens the entire U.S. citrus industry, and globally is rapidly spreading. Florida’s citrus production is 90% sweet orange, which is quite sensitive to HLB. The heavy reliance on sweet orange for Florida citrus production makes the industry especially vulnerable to diseases that are damaging to this type of citrus. Furthermore, 90% of Florida oranges are used in producing orange juice that is defined by a federal regulation known as the “orange juice standard”, specifying that at least 90% of “orange juice” must be derived from *Citrus sinensis*. Genomic analyses definitively reveal that sweet orange is not a true species, but just one of many introgression hybrids of *C. reticulata* and *C. maxima*, with phenotypic diversity resulting from accumulated mutations in this single hybrid, the “sweet orange”. No other fruit industry is limited by law to such a narrow genetic base. Fortunately, there are new citrus hybrids displaying reduced sensitivity to HLB, and in some cases they produce juice, alone or in blends, that consumers would recognize as “orange juice”. Reconsidering current regulations on orange juice standards may permit use of such hybrids in “orange juice”, providing greater latitude for commercialization of these hybrids, leading to higher-quality orange juice and a more sustainable Florida orange juice industry.

## Introduction

Huanglongbing (HLB) has devastated the citrus industry in Florida, which declined in total production by 80% between 2003–2004 and 2017–2018^1^. HLB threatens the entire U.S. citrus industry, and is becoming more widespread globally. *Candidatus* Liberibacter asiaticus (*C*Las) is the bacterium associated with HLB in the U.S., and is vectored by *Diaphorina citri*, the Asian citrus psyllid (ACP). It can take many months for symptoms to become evident after an initial infection. As symptoms progress, most infected trees become less vigorous, with root decline, thinning canopies and abnormal leaves, culminating in yield loss and diminished fruit quality^2,3^. HLB was first positively identified in Florida in 2005, and it is now ubiquitous in most citrus-growing areas of the state. HLB was confirmed in Texas and California in 2012: it is increasingly widespread in Texas^4^, and >1500 HLB-affected dooryard trees have been identified and removed in California^5^.

Florida citrus growers primarily manage HLB using production practices intended to reduce stress in infected trees and most still use frequent insecticide applications for vector control. Production costs for processing oranges increased ~2.5-fold (2015–2016 data for the Central Florida Ridge^6^) compared with pre-HLB costs^7^. Growers report that even with these costly practices, overall citrus production per acre has declined an average of 41%^8^. It was estimated in 2017^9^ that the total cumulative economic impact of HLB on Florida citrus was -$US 4.6 billion with a loss of 32,000 job-years. Further, two decades ago there were 30 major citrus-processing plants, but currently only seven remain^10^. If this trend is not reversed, the remaining processing plants will not have enough fruit to maintain profitability and will be out of business. The loss of this valuable infrastructure cannot be easily recovered.

Generally, of the two most widely planted sweet orange cultivars, ‘Valencia’ production is less affected than ‘Hamlin’ (Florida citrus growers, personal comm.). Enhanced production practices can provide good quality juice, but some trailer-loads of fruit have not met standards and could not be processed due to off-flavors. Juice extracted from fruit harvested from HLB-affected trees has higher levels of bitter compounds, such as limonin^11^. The blending procedure for processors, to provide a consistent high-quality product, has become more challenging and expensive as delivered fruit show more variability in quality, and the harvest/processing season is interrupted due to reduced supplies of fruit. There is evidence that HLB-affected trees are much more susceptible to stresses and other diseases, as well. Major stresses such as freeze events and hurricanes may reduce yields further and compromise trees much more than was observed with healthy trees. Growing HLB-resistant or -tolerant citrus cultivars is considered to be the most plausible solution to achieve sustainability of citrus production where HLB is endemic^2^.

## A monoculture of sweet orange

Florida’s citrus production is 90% sweet orange, 8% grapefruit, with the remainder mainly mandarins and mandarin hybrids^1^. Unfortunately, all of the major Florida citrus cultivars are quite sensitive to HLB. The heavy reliance on sweet orange for Florida citrus production makes the industry especially vulnerable to diseases that are damaging to this type of citrus, as is the case of HLB. A fundamental concept of plant pathology is that a narrow genetic base exposes agriculture to great risk when a susceptible crop is exposed to a new epidemic (reviewed in ref. ^12^). A striking modern example was the loss of 15% of the US and Canadian maize (*Zea mays*) crop in 1970–1971 from southern corn leaf blight. Greater than 85% of plants had a single cytoplasmic genotype conferring male sterility in female lines used for hybrid seed production. High susceptibility to the T-strain of the pathogen *Bipolaris maydis* resulted in losses approaching 100% on some farms (reviewed by Bruns^13^). More devastatingly, the Irish potato famine of 1845–1849 was exacerbated by primary reliance on a potato variety, the ‘Lumper’, which was highly susceptible to late blight induced by *Phytophthora infestans*, and resulting crop losses contributed to the death of more than a million people^14^.

Global awareness of the dangers of monoculture has recently received heightened public attention with the launch of the initiative “One Planet Business for Biodiversity”. This initiative was launched by a global consortium of large corporations such as Google, Danone, Nestle, MARS, etc. to promote crop diversity^15^.

## Combatting disease with resistance

There are several examples where a change in planting material has provided a solution to an otherwise intractable disease. As one prominent example, the banana (*Musa* sp.) industry was devastated by Panama disease (caused by *Fusarium oxysporum* f. sp. *cubense*), and the solution was to transition from the susceptible cultivar ‘Gros Michel’ to the resistant ‘Cavendish’. New races of the Panama disease pathogen have overcome the resistance of ‘Cavendish’ and new tolerant cultivars are in development (reviewed in ref. ^16^). Resistant plants have provided solutions for numerous crops and their diseases, such as potato^14^ and maize^13^ from our earlier examples of epidemics.

A list was compiled by the Sainsbury Laboratory (Norwich) of the most critical plant epidemics (epiphytotics) of the 20th century^17^. It is proposed that the best solutions for most of these epidemics are identification and/or development of planting material with genetic resistance or tolerance. The only historical plant epidemic for which the ultimate solution was chemical, and not genetic, was an epiphytotic from the nineteenth century: downy mildew *(Plasmopara viticola*) in grape (*Vitis vinifera*) was controlled through foliar application of copper compounds^18^. The wine industry’s dependence on well-known asexually propagated grape cultivars largely explains this exception, and grape hybrids have been developed which are resistant to this disease^19^.

## Tolerance to huanglongbing in citrus

It is vital that HLB-tolerant alternatives to current scion cultivars be identified and implemented. HLB-tolerant/resistant solutions through biotechnology (i.e., introduction or modification of targeted genes) could permit continued use of current cultivars with traits otherwise identical to the types now grown; however, deregulation will likely be a lengthy and expensive process, and consumer acceptance is uncertain. It is possible that natural or induced mutants may provide “true” sweet oranges with enhanced performance where HLB is endemic, but this has not yet been documented in the 75 years that HLB has been studied. Future opportunities will require a shift to new cultivars to help sustain the stream of juice needed for our customers.

Fortunately, it appears that there is resistance or field tolerance to HLB within citrus and citrus relatives that has been described over many years prior to finding HLB in the U.S. In Florida, several released cultivars appear to have commercially useful levels of HLB tolerance, and these are predominately mandarin in their pedigrees. ‘LB8–9’ Sugar Belle® (Gmitter^20^); and ‘Bower’ are especially noteworthy for their HLB tolerance, as is a new USDA release ‘US SunDragon’ (Stover unpublished). Several hybrids and previously released cultivars also seem to have useful HLB tolerance, but have not yet been widely planted. More than 30,000 diverse new interspecific hybrids have been created in the last 10 years, and some are expected to display significant improvement in yield and fruit quality because of HLB tolerance, as well as other current pests and diseases, and/or new threats (i.e., new diseases, climate change, salinity, etc.), through leveraging the extensive genetic diversity available in the genus.

## Sweet orange and its origins

The sweet orange is among the most widely planted fruit trees in the world^21^. The industries most focused on sweet orange are Brazil, which produced sweet orange on 97% of its 995,000 citrus acres (2017 data^22^) and Florida, USA with 90% of its 455,000 citrus acres in sweet orange (2017 data^1^). Its distinctive flavor and aroma characteristics are greatly appreciated by consumers, and a handful of sweet orange cultivars dominate many of the world’s citrus industries. Processors, packers, and growers have invested considerable resources to optimize quality of resulting products.

Sweet orange had been considered a distinct species, *Citrus sinensis*, since its seedlings all share similar traits, and there is some phenotypic diversity in fruit traits such as season of maturity, shape, size, flavor and aroma, and pigmentation. Attempts to use sweet orange as a seed parent to hybridize with other citrus documented that similarity of seedlings is due to a high degree of apomixis, via nucellar embryony^23^. Molecular marker studies using isozymes, RAPDs, SSRs, etc., indicated that cultivars of sweet orange are genetically almost identical, despite some phenotypic differences (e.g. refs. ^24,25^) that arose by genic or chromosomal mutations.

**Fig. 1:**
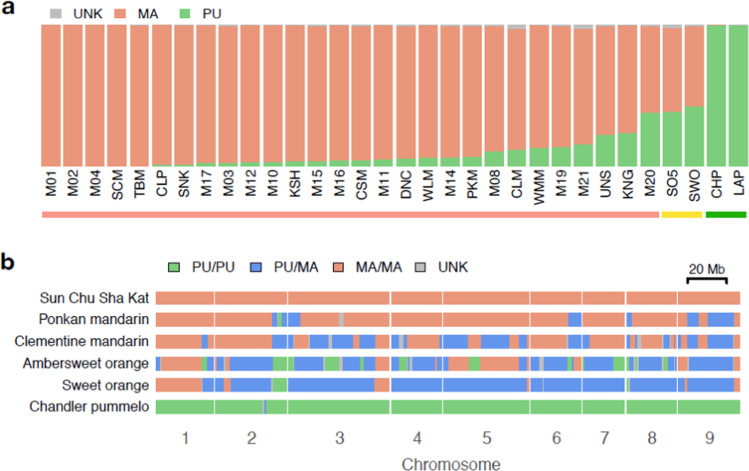
Pummelo introgression in mandarins and oranges. **a** Pummelo and mandarin allelic proportion in the genomes of mandarins, oranges and pummelos, underlined by horizontal bars in red, yellow and green, respectively. The two progenitor species are denoted by PU = *C. maxima* (green) and MA = *C. reticulata* (red). The three-letter codes correspond to cultivar names and can be found in ref. ^27^ (derived from Fig. 2a). Especially noteworthy for this paper: SWO = sweet orange (*C. sinensis*) and SO5 = ‘Ambersweet’ orange-like hybrid. **b** Local genetic ancestry of some representative cultivars (^27^; derived from ED Fig. 2a). The chromosome designations are based on the haploid Clementine reference sequence^26^

Genomic studies have shown with great clarity that the concept of sweet orange as a distinct biological species, *Citrus sinensis*, is flawed. As is the case for many commercial citrus types, these studies show that sweet orange arose from complex interspecific introgression (^26,27^; Fig. 1a). It appears that all sweet oranges likely arose from a single individual ancestor, derived solely from the introgression of two ancestral true species *C. maxima* (Pummelo) and *C. reticulata* (Mandarin). Sequence analysis permits inferences on detailed pedigree based on hetero- vs. homozogosity across paired chromosomal fragments (Fig. 1b). Wu et al.^26^ proposed that the sweet orange pedigree is consistent with [(*C. maxima* × *C. reticulata*) × (*C. maxima*)] × mandarin (*C. reticulata)*, with some *C. maxima* admixture in the largely *C. reticulata* mandarin pollen parent. Cultivar variants in sweet orange then resulted from mutations within this original complex hybrid, which accumulated over time to produce the range of sweet orange cultivars that exist today.

The near-isogenic nature of sweet orange cultivars is especially important since some citrus industries rely so heavily on this narrow genetic base, forming monocultures. Breeding more diverse cultivars offers a compelling solution, but existing market expectations and associated regulations are substantial impediments to implementation of that solution.

## Orange juice standards

“Orange juice” is the iconic product of the Florida citrus industry, recognized by people around the world, and in 2015–2016 US consumption was 2.2 gallons per capita^28^. In an effort to protect quality and Florida’s growers, the Florida citrus industry led an effort in the 1970s to codify “orange juice” in US law, resulting in 21 CFR 146.135:

“Orange juice is the unfermented juice obtained from mature oranges of the species *Citrus sinensis*”, with additional designation that no more than 10% other juice from *C. reticulata* or *C. reticulata* hybrids can be used. The code was further modified in 1992 to allow a single sexually derived orange-like hybrid, ‘Ambersweet’, to be considered legally as a sweet orange^29^.

While there are a few industries that have chosen to focus primarily on a single cultivar (e. g. ‘Montmorency’ tart cherry; ‘Kerman’ pistachio; ‘Wonderful’ pomegranate), no other tree fruit industry is so narrowly defined by law to a single genotype. In contrast, apple juice can include juice from any combination of the 7500 recognized apple cultivars, with more than 100 cultivars commonly grown in the USA^30^. These cultivars include some, like ‘Liberty’^31^, with disease resistance derived from interspecific hybridization with *Malus floribunda*.

The current US regulation defining orange juice as being almost exclusively from *C. sinensis* locks the industry into a very narrow range of genetic diversity, leaving the Florida citrus industry extraordinarily vulnerable to epidemics when diseases or pests emerge to which sweet orange is highly susceptible, such as HLB. The modification of the code to include ‘Ambersweet’, however, sets precedent for the inclusion of other orange-like hybrids, following lengthy validation to demonstrate their close similarity in taste and appearance with sweet oranges. Other regulatory changes in the orange juice standard of identity should provide even greater opportunities for evolution and improvement of this important industry.

The orange juice market is global, with the USA exporting ~10% of its production, and importing product essentially equal in volume to domestic production^32^. Some countries have followed the US regulations in defining orange juice standards, which are in alignment with the Codex Alimentarius, while other entities, such as the European Union only permit use of the term orange juice if it is 100% *C. sinensis*^33,34^. Any changes in US OJ standards will likely conflict with the requirements of some other citrus importing and exporting countries, requiring changes to the Codex standard and certain national standards to truly represent an international standard.

## Sweet orange-like hybrids

To provide greater genetic diversity, citrus breeders have attempted to create improved sweet orange types for decades. In 1989 USDA/ARS released ‘Ambersweet’ which was noteworthy in its resemblance to sweet orange. Chemical and organoleptic evaluations resulted in the official acceptance of ‘Ambersweet’ as a ‘sweet orange’^23,29^, and it was widely planted in Florida, but suffered from low productivity^35^. However, a new generation of sweet-orange-like hybrids, produced by the University of Florida and USDA citrus breeding programs, are under evaluation, some of which also include a small proportion of *Poncirus trifoliata* in their pedigrees. Volatile aromatic profiles from some of these hybrids are even more like sweet orange than are those from ‘Ambersweet’^36^. However, few growers are likely to plant commercial acreage, until the fruit have a clear market. Therefore, one approach to encourage deployment of new HLB-tolerant cultivars may be to develop a procedure and establish criteria for accepting new hybrids as sweet oranges, which could then be codified into law, on a case-by-case basis.

The ‘Ambersweet’-precedent for classifying citrus hybrids as “orange” is based on the aromatic volatile profile since the nonvolatile flavor compounds (sugars, acids, flavonoids, and limonoids) are similar for most edible citrus types^36,37^. Volatile profiles in mandarins and mandarin hybrids are largely diverse^38^, but some of them contain total volatile concentrations and profiles comparable to oranges, and have the potential to be classified as “sweet orange” based on their aroma profile^36,39^. Volatiles such as ethyl butyrate, other ethyl esters, valencene, and other sesquiterpenes may help classify hybrids as orange. However, trace-level volatiles that are often associated with mandarins and their hybrids, but not oranges, are δ-3-carene, methyl N-methylanthranilate, thymol, and its methyl ether. Feng et al.^40^ focused on odor activity values (OAV) and used aroma extract dilution analysis (AEDA) with a trained sensory panel to demonstrate that ethyl butonate, ethyl 2-methylbutonate, octanal, decanal, and acetaldehyde were essential for orange-like aroma, whereas linalool, octanal, α-pinene, limonene, and (E, E)-2,4-decadienal were critical components for mandarin-like flavor. These compound profiles may be used as indicators to differentiate those hybrids producing fruit that are more or less sweet orange-like.

Unfortunately, the ‘Ambersweet’ model may require detailed evaluation of each potential new “sweet orange” and case-by-case legal procedures for inclusion in regulations. Therefore, a more generic definition for the orange juice standard of identity is desirable, and it would include citrus hybrids with fruit and juice traits similar to sweet orange. Otherwise, citrus growers will be reluctant to plant new HLB-tolerant hybrids, suppressing innovation and hindering a breeding solution to HLB.

## Expanding the definition of orange juice

The change in regulations that would permit greatest utilization of HLB-tolerant planting stock would be to focus on aroma and flavor in defining orange juice. As described above, the scientific community no longer views *C. sinensis* as a true species. It is only one of an infinite number of possible recombinations of the two ancestral species (other examples in Fig. 1, along with some pure species accessions). So, reliance on sweet orange alone for most Florida orange juice is not supported by genome science and taxonomic systems, but rather is solely a regulatory classification. Some of the HLB-tolerant “mandarin hybrids” developed by breeding programs produce juice chemically and organoleptically resembling true sweet orange juice, while others can contribute to blends indistinguishable from orange juice. It seems advisable that discussions focus more on the quality characteristics of the resulting juice product rather than the exact genetic makeup of the plant producing the juice. Clearly, this must occur within the context of producing an excellent, reliable product which is consistent throughout all markets and months. Consumer focus group studies could further assess the acceptability of orange-like or high-quality “citrus” juices.

The regulatory language being considered to allow broader cultivar inclusion might read something like:

“Orange juice is the unfermented juice obtained from mature fruit of the sweet orange (conforming to the genotype known as *Citrus sinensis)*, and/or from fruit with an ancestral interspecific pedigree similar to that of the sweet orange, and with color, flavor and organoleptic properties typical of sweet orange.”

This definition would include ‘Ambersweet’ and other hybrids with sweet orange-like traits and predominately derived from *C. reticulata* and *C. maxima* (Fig. 1).

All these discussions focus on defining “orange juice” to help sustain the processing citrus industry. No regulation prevents use of any citrus in producing a “citrus juice”, however, consumer recognition of and expectations for “orange juice” are of incalculable value. This product equity supports a good-faith effort to expand “orange juice” to help maintain quality and volume, which will benefit consumers as well as producers. Ultimately, but inherently longer-term, even greater benefit to the industry and consumers may arise from broadening products well beyond orange juice, focusing simply on high-quality, excellent tasting juice that will appeal to consumers. In our experience, juice made from HLB-tolerant mandarin hybrids either alone, or mixed together, or combined with sweet orange juice are preferred by many and sometimes most consumers. In recent reports, a blend of 50% ‘LB8–9’ Sugar Belle® and 50% sweet orange juice was scored almost the same as 100% juice from each of four sweet orange cultivars tested^41^. In another study, a 10% Sugar Belle® with 90% ‘Valquarius’ sweet orange and a 50:50 blend were preferred in consumer tests over a 100% sweet orange commercial juice product^42^.

A new USDA-NIFA project (Accelerating implementation of HLB-tolerant hybrids as new commercial cultivars for fresh and processed citrus; with PIs from USDA, University of Florida and University of California Riverside) aims to characterize juice and juice blends from HLB-tolerant citrus, with the expectation that some cultivars alone or in combination will provide customers with a highly desirable orange juice-like product and provide the juice stream needed for a sustainable Florida citrus industry. About 30 hybrids have been extensively analyzed, and their potentials for use in blends resembling sweet orange juice are being evaluated. New chromatography profiling software will be applied to determine similarities in aroma volatile profiles of what is now called orange juice vs. hybrid juices and citrus juice blends. Taste panels are also being used to develop data on consumer liking of hybrid juice and blends compared to orange juice standards.

## Expanding the other juice category

A simpler approach to expanded use of HLB-tolerant planting stock, could be to increase the current “10% other juice” regulation, to permit a greater proportion of other cultivars to be used in orange juice. Examples from the wine industry are supportive of such an approach: varietal designation in US wine only requires 75% composition from the designated variety, with the exception that only 51% is required for designation as a *Vitis labrusca* variety^43^.

Existing regulatory language permitting use of *Citrus reticulata* or *Citrus reticulata* hybrids for the “other juice”, combined with increasing the percentage permitted, would facilitate broad acceptance of HLB-tolerant material as most citrus under consideration meet this taxonomic definition. This could potentially include selections with *Poncirus* in the pedigree, which also can be useful for HLB tolerance, provided they have no negative impact on flavor and absence of distinctive aromatic compounds to be used in blends.

## In summary

1. Sweet orange is just one of many hybrids derived from introgression of the genomes of *C. reticulata* and *C. maxima*. Reliance on a single chance hybrid is unprecedented in any other fruit commodity and exposes the orange juice industry to devastating epidemics, even beyond HLB, due to the very narrow genetic base.
2. Many other hybrids have been identified with greater tolerance to HLB than sweet orange, that are either solely or largely derived from the same two parental species as sweet orange.
3. Some of these hybrids produce juice closely resembling sweet orange juice, when used alone, when blended together, or used in blends with sweet orange.
4. Some produce very high-quality juice that is excellent, but notably different from orange juice.
5. Therefore, the following non-mutually exclusive approaches are being considered to enhance sustainability of the orange juice processing industry:
  - Developing a procedure and establishing criteria for accepting new hybrids as “orange juice” components, and incorporate them into a new standard of identity.
  - Adjusting the “10% rule” to permit a greater proportion of juice from other hybrids, with the continued goal of generating a consistent product that will meet quality expectations for “orange juice”.
  - Consider expanding production of other high-quality citrus juices that do not necessarily duplicate OJ as stand-alone (like tangerine juice already on the market), or as citrus juice blend products.
